# Protecting vision with intraoperative visual evoked potentials and tractography in transcortical brain tumor surgery

**DOI:** 10.22336/rjo.2024.56

**Published:** 2024

**Authors:** Ioannis Mavridis, George Tokas, Efstratios-Stylianos Pyrgelis, Theodossios Birbilis

**Affiliations:** 1 Department of Neurosurgery, School of Medicine, Democritus University of Thrace; University General Hospital of Alexandroupolis, Alexandroupolis, Greece; 2 1st Department of Neurology, School of Medicine, National and Kapodistrian University of Athens; Eginition Hospital, Athens, Greece

**Keywords:** brain tumor surgery, intraoperative neuromonitoring, optic radiation, visual pathways, visual evoked potentials, ERG = electroretinogram, GCS = Glasgow coma scale, IONM = intraoperative neuromonitoring, LED = light-emitting diodes, MRI = magnetic resonance imaging, PET = positron emission tomography, TSS = transsphenoidal surgery, VEPs = visual evoked potentials

## Abstract

**Objective:**

Intraoperative neuromonitoring (IONM) is nowadays a gold standard during brain tumor resections, but visual function mapping is less frequently performed in clinical practice. This article aims to report two transcortical brain tumor surgery cases affecting optic radiation, where the application of intraoperative visual evoked potentials (VEP) combined with tractography was beneficial to protect the patients’ vision.

**Methods:**

Two patients with brain tumors compressing the left posterior visual pathways underwent surgery under general anesthesia using IONM and VEP with neurologic improvement and preservation of vision.

**Results:**

VEP is beneficial in the surgery of intra-axial lesions affecting the posterior visual pathways (optic radiation, visual cortex) and parasellar lesions involving the anterior visual pathways (chiasm). They can also be effectively combined with other mapping methods such as tractography.

**Conclusions:**

According to our experience, IONM with VEPs and neuronavigation with tractography protect visual function in transcortical approaches to resecting tumors near the optic radiation and should be considered a standard monitoring method for such operations.

## Introduction

Preoperative studies with magnetic resonance imaging (MRI), and often functional assessments with positron emission tomography (PET) and functional MRI, are essential to recognize and preserve eloquent cortex in intracranial tumor surgery [1]. Although cortical functional areas and fiber tracts can be localized preoperatively by probabilistic anatomical tools, functional integrity mapping by neurophysiology is essential [2]. Intraoperative neuromonitoring (IONM) is nowadays a gold standard during brain tumor resections and other intracranial procedures [1] and is particularly useful while operating in functionally vital brain areas. It requires careful coordination between neurosurgeons, neurophysiologists, and anesthetists [3].

IONM methods include somatosensory evoked potentials, transcranial motor evoked potentials, visual evoked potentials (VEPs), brainstem acoustic evoked responses, stimulation mapping of motor cortex and speech areas, and stimulation of cranial nerves [3]. Intraoperative mapping represents the main means to avoid neurologic damage as it can be performed during the entire surgical procedure to prevent direct damage to brain tissue or indirect damage due to ischemia or edema (e.g.: from brain retraction) [4]. Regarding optic pathways’ protection, however, visual function mapping is less frequently performed in clinical practice [5].

VEPs are scalp-recorded visual cortex potentials extracted from the electroencephalogram by signal averaging. There are several methods of recording VEPs, and the recording electrode is usually placed at the midline of the occipital area. VEPs are used to quantify the functional integrity of the visual pathways, and can therefore be affected by any abnormality (damage/irritation) of these fibers. In sedated patients, flashes of light from a strobe flash or an array of light-emitting diodes (LED) stimulate the eye [6]. Intraoperative VEPs are not routinely considered an integral part of IONM and their use is usually suggested for tumors nearby or involving the visual pathways. In this context, this article aims to report two difficult transcortical brain tumor surgery cases affecting optic radiation, in which the application of intraoperative VEP, combined with tractography, was beneficial in protecting the patients’ vision.

## Methods

### Case 1

**Fig. 1:**
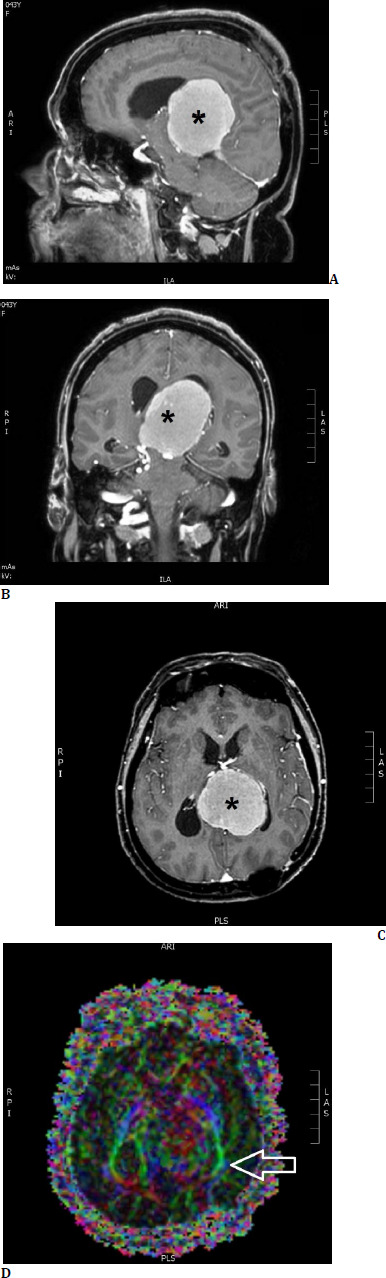
Brain MRI of our first patient, T1-weighted sequence with intravenous contrast, showing a large almost centrally located falcotentorial meningioma (*). **A**. sagittal section; **B**. coronal section; **C**. transverse section; **D**. tractography showing distortion of the fibers of the left optic radiation (arrow)

The first case was a 43-year-old woman, who presented with numbness of her right hemibody, generalized weakness, headaches, dizziness, and illusions, due to a known previously biopsied large falcotentorial meningioma (World Health Organization Grade I) (**Fig. 1**). Physical examination showed a Glasgow coma scale (GCS) score of 15/15, episodes of left hemianopia, episodes of abnormal gaze coordination during horizontal eye movements and mild left facial nerve palsy. Cerebellar dysfunction was also detected, with imbalance during upright position and unsteady walking. The patient also presented a positive right Barré sign and mild muscle weakness (4/5) of the right upper limb.

### Case 2

**Fig. 2:**
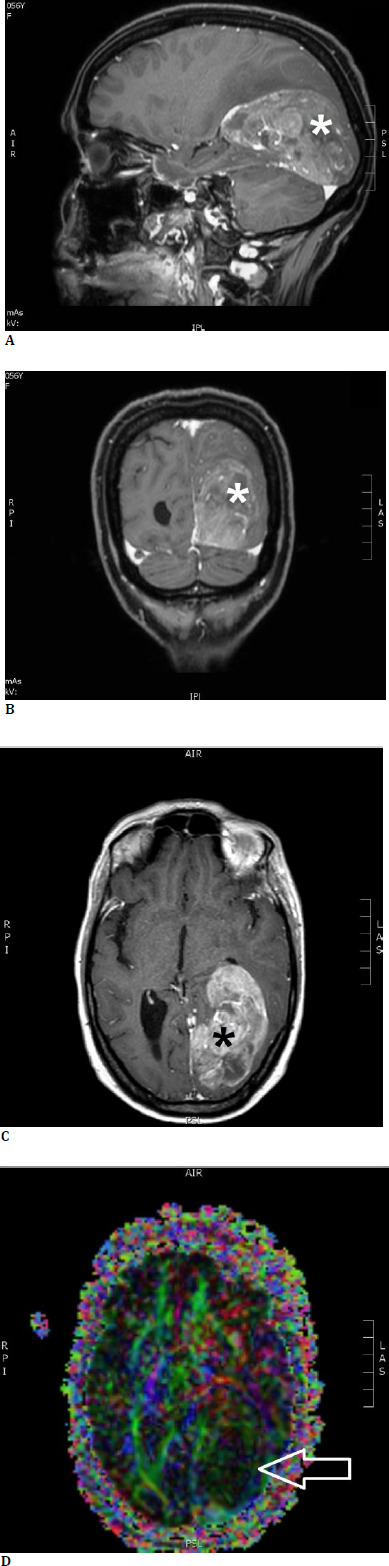
Brain MRI of our second patient, T1-weighted sequence with intravenous contrast, showing a giant left temporooccipital metastasis (*) from lung carcinoma. **A**. sagittal section; **B**. coronal section; **C**. transverse section; **D**. tractography showing distortion of the fibers of the left optic radiation (arrow)

The second case, a 56-year-old woman, presented to the Emergency Department, following a psychiatric evaluation from a private practitioner, with symptoms of mild dementia, headaches, and balance disorder. Her GCS score was 13/15, she was disorientated to time and dysphasic, with mild weakness of the lower extremities and unsteadiness. Her brain MRI scan revealed a giant left temporooccipital mass (**Fig. 2**) and suspicion of another minimal one in the cerebellar vermis. A computed tomography scan of her chest also showed a large mass of the lower lobe of the left lung, which was the primary tumor of the patient (neuroendocrine carcinoma).

## Results

### Surgery with intraoperative VEP and neuronavigation including tractography

**Fig. 3:**
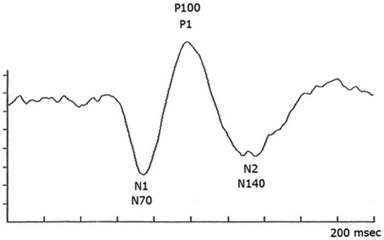
Normal intraoperative VEP waveform; horizontal intervals are set at 20 msec; N70, P100, N140: separate phases of the waveform

Both patients underwent surgery (subtotal resection) under general anesthesia in prone position, using neuronavigation with tractography, intraoperative ultrasound, IONM, and VEP (**Fig. 3**). A left parietal craniotomy with transcortical parietal approach (with risk of damaging the optic radiation) was performed in the first patient and a left occipital craniotomy with transcortical occipital approach (with risk of damaging the optic radiation and visual cortex) in the second. Both patients tolerated surgery well, with full recovery and no visual or neurologic deficits at discharge (a week later).

Regarding the applied VEP system (Cadwell Industries, Inc., Kennewick, WA, USA), 8 cm LED visual stimulators were used, with a pattern reversal checkerboard stimulator and 6.4 mm red and black square. The LED goggles were carefully applied to exclude external light from the stimulation field, allowing flash pattern stimulus selectable for right, left, or both eyes. The flash stimulator was programmed at flash rates between .5-15 Hz, 0.72 J/flash.

## Discussion

### Posterior visual pathways (intra-axial tumors)

The sensitivity and specificity of VEP in visual dysfunction detection have been reported close to 100% given that the parameters of anesthesia meet the appropriate requirements [3]. Maryashev et al. (2020) [7] reported intraoperative mapping with cortical VEP during occipital tumor resection. They also found it valuable in reducing the risk of damage to the visual cortex and visual pathways with subsequent postoperative visual impairment [7]. As presented above, our experience confirms their conclusions.

In their interesting study of 29 patients with resected intra-axial brain lesions, Gutzwiller et al. (2018) [8] aimed to determine the ability of intraoperative VEP to detect visual field changes. Intraoperative VEPs were performed with simultaneous electroretinogram (ERG) recording, with protection from the operating room lights and white LED. The alarm criterion they set (i.e., ≥ 20% decrease in amplitude of the VEP P100 wave) identified 100% of cases with changes more severe than just a discrete quadrantanopia or deterioration of an existing quadrantanopia. The absence of alarm was associated with postoperative visual deficits absence in 94% of cases. The authors underlined that intraoperative VEP allowed at minimum the detection of new quadrantanopia and suggested this technique’s standardization [8].

### Anterior visual pathways (parasellar tumors)

Vision worsening is a serious potential complication of transsphenoidal surgery (TSS), usually for tumors of the sellar region. These patients represent another category in whom intraoperative VEP monitoring is a reproducible and effective technique for predicting postoperative visual field defects. VEP amplitude decrease is considered a warning sign of injury to the anterior optic apparatus that justifies halting surgical manipulation [9]. Kamio et al. (2014) [10] adapted a high-power light-stimulating device with ERG to ascertain retinal light stimulation, to evaluate the usefulness of intraoperative VEP monitoring in TSS (33 patients). They found this device reliable and feasible in preserving visual function (≥ 50% decrease in amplitude was correlated with postoperative vision deterioration) [10].

Miyagishima et al. (2019) [11] applied VEP monitoring using LED during extended endoscopic endonasal TSS for craniopharyngiomas. If the VEP changed and became unstable, the surgeon was informed and stopped the surgical manipulation of the optic chiasm awaiting electrophysiologic recovery. Vision was improved after surgery in all (seven) patients and the authors supported VEP monitoring as the only way to test visual function during TSS, considering it helpful in the extended endoscopic endonasal TSS approach (which requires dissection between the optic nerve and tumor) [11]. Following the previous authors, Toyama et al. (2020) [12] adapted a high-power LED stimulator with ERG and used a black shield patch and braided codes to obtain reproducible VEP amplitudes (20 patients). They observed no postoperative visual impairment by temporarily halting their surgical manipulation when VEP deteriorated. They thus suggested VEP monitoring as a warning method to avoid postoperative visual dysfunction and recommended VEP as a routine aid in TSS [12].

### Combined and alternative intraoperative visual monitoring methods

Intraoperative navigation using tractography and visual pathways monitoring with VEP, as used in our patients, is reliable in monitoring visual function and helpful in neurosurgical planning close to the visual pathways [13] and may allow maximal resection while preserving vision in patients with tumors adjacent to the optic radiation [14]. VEP can also be compared to neurosurgical preplanning methods, such as sites of functional PET activation during the preoperative performance of visual tasks [4]. Moreover, intraoperative sodium fluorescein combined with VEP helps maximize tumor resection and predict postoperative functional outcomes in anterior visual pathway tumor surgery [15].

Shahar et al. (2018) [14] evaluated the value of visual pathway mapping in 18 patients undergoing tumor resection via awake craniotomy. Direct cortical VEP recording, subcortical recordings from the optic radiation, and subcortical stimulation of the latter were used intraoperatively to assess the visual function and proximity of the lesion to the optic radiation fibers. Interestingly, they found a positive correlation between subcortical threshold stimulation intensity within the distance from the optic radiation. Subcortical recordings from the optic radiation demonstrated a typical VEP waveform in 77%, which was present only when recordings were obtained within 10 mm from the optic radiation. Postoperative visual field deterioration (39%) was associated with a < 8 mm distance between the tumor and optic radiation. Additionally, no association was found between preoperative visual field status and baseline presence of cortical VEP [14].

Direct electric stimulation, instead of VEP, can also be intraoperatively used to assess visual function. Although less popular than VEP, it could have better clinical outcomes (hemianopia decreased frequency) [16]. Recently, Santos et al. (2022) [16] described an intraoperative central and peripheral image task that maps optic radiations during brain tumor resection in three patients. Optic radiations were identified in all patients and preserved in all but one (with a greater extent of resection) [16]. Finally, Sobottka et al. (2013) [5] showed that the visual cortex can be intraoperatively mapped with a contact-free optical camera system. Activity maps could be reproducibly computed by blood volume-dependent signal changes of the exposed visual cortex detection [5].

## Conclusions

We reported our experience of two challenging brain tumor surgery cases affecting the left optic radiation, in which the application of intraoperative VEP was beneficial to preserve the patients’ visual function. VEPs are particularly useful in the surgery of intra-axial lesions affecting the posterior visual pathways (optic radiation, visual cortex), and parasellar lesions involving the anterior visual pathways (chiasm). They can also be effectively combined with other mapping methods such as tractography. Based on our findings, we suggest IONM with VEP and neuronavigation with tractography as a standard combination for intraoperative monitoring in transcortical approaches to resecting tumors affecting the optic radiation to protect the patient’s vision.
